# Neural Correlates of Abnormal Temporal Discrimination in Unaffected Relatives of Cervical Dystonia Patients

**DOI:** 10.3389/fnint.2019.00008

**Published:** 2019-03-12

**Authors:** Shruti Narasimham, Eavan M. McGovern, Brendan Quinlivan, Owen Killian, Rebecca Beck, Sean O’Riordan, Michael Hutchinson, Richard B. Reilly

**Affiliations:** ^1^Trinity Centre for Bioengineering, Trinity College Dublin, University of Dublin, Dublin, Ireland; ^2^School of Engineering, Trinity College Dublin, University of Dublin, Dublin, Ireland; ^3^School of Medicine and Medical Science, University College Dublin, Dublin, Ireland; ^4^Department of Neurology, St. Vincent’s University Hospital, Dublin, Ireland; ^5^School of Medicine, Trinity College Dublin, University of Dublin, Dublin, Ireland

**Keywords:** cervical dystonia, temporal discrimination, resting state fMRI, dual regression, connectivity

## Abstract

**Background:** An abnormal temporal discrimination threshold in cervical dystonia (CD) is considered to be a mediational endophenotype; in unaffected relatives it is hypothesized to indicate non-manifesting gene carriage. The pathogenesis underlying this condition remains unknown. Investigation of the neural networks involved in disordered temporal discrimination may highlight its pathomechanisms.

**Objective:** To examine resting state brain function in unaffected relatives of CD patients with normal and abnormal temporal discrimination. We hypothesized that the endophenotype, an abnormal temporal discrimination, would manifest as altered connectivity in relatives in regions associated with CD, thereby illuminating the neural substrates of the link between temporal discrimination and CD.

**Methods:** Rs-fMRI data was analyzed from two sex- and age-matched cohorts: 16 unaffected relatives of CD patients with normal temporal discrimination and 16 with abnormal temporal discrimination. Regional and whole brain functional connectivity measures were extracted via Independent Component Analysis (ICA), Regional Homogeneity (ReHo), and Amplitude of Low Frequency (ALFF) analyses.

**Results:** Our ICA analysis revealed increased connectivity within both the executive control and cerebellar networks and decreased connectivity within the sensorimotor network in relatives with abnormal temporal discrimination when compared to relatives with normal temporal discrimination. The ReHo and ALFF analyses complimented these results and demonstrated connectivity differences in areas corresponding to motor planning, movement coordination, visual information processing, and eye movements in unaffected relatives with abnormal temporal discrimination.

**Conclusion:** Disordered connectivity in unaffected relatives with abnormal temporal discrimination illuminates neural substrates underlying endophenotype expression and supports the hypothesis that genetically determined aberrant connectivity, when later coupled with unknown environmental triggers, may lead to disease penetrance.

## Introduction

The sensory temporal discrimination threshold (TDT) is defined as the shortest interval at which two sequential sensory stimuli are perceived as asynchronous (Lacruz et al., 1991); stimuli may be visual, tactile, or auditory. The neural basis of time processing has been examined using tasks such as frequency discrimination, time estimation, and temporal order judgment (Lacruz et al., 1991; Pastor et al., 2004). Evidence from lesion and neurophysiological studies suggests that temporal discrimination involves the time locked activation of both subcortical and cortical neural networks (Handy et al., 2003). However, the precise neural circuitry involved in temporal discrimination remains unknown. Furthermore, the TDT has been shown to vary physiologically by age (Ramos et al., 2016) and sex (Butler et al., 2015).

Temporal discrimination is disordered in a number of basal ganglia diseases including adult-onset dystonia. Dystonia is a hyperkinetic movement disorder characterized by sustained or intermittent muscle contractions causing abnormal movements and posture (Albanese et al., 2013). Cervical dystonia (CD) is the most common phenotype of adult onset isolated focal dystonia and is considered to be an autosomal dominant disorder with markedly reduced penetrance (Waddy et al., 1991).

Recent studies suggest that an abnormal TDT is an endophenotype of adult onset focal dystonia. Abnormal TDTs have been found in CD, with high sensitivity and specificity, and in up to 52% of unaffected relatives of patients with CD (Bradley et al., 2009; Kimmich et al., 2014). It is hypothesized that an abnormal TDT is a *mediational* endophenotype in CD and, as such, is considered a subclinical marker of gene carriage, not altered by disease penetrance or expression. A mediational endophenotype shares common pathogenetic mechanisms with the phenotype; study of the endophenotype may illuminate mechanisms which are not obvious from the phenotype (Hutchinson et al., 2013). Despite the potential importance of temporal discrimination as a marker of disordered sensory processing in the pathophysiology of CD, the neural network function underlying abnormal temporal discrimination in CD patients and their unaffected relatives is poorly understood.

The heterogeneity of phenotypes makes it imperative to study each phenotype and its associated endophenotype in patients as well as unaffected relatives (Battistella et al., 2015). Some studies in the past have explored functional changes in unaffected relatives of dystonia patients. For example, regional metabolic changes (Carbon et al., 2004) were observed in non-manifesting DYT1 (an autosomal dominant, early onset, generalized dystonia) mutation carriers. Resting state functional MRI (Rs-fMRI) studies have primarily been used to demonstrate brain function abnormalities between CD patients and controls (Delnooz et al., 2013; Li et al., 2017). Because mutated genes (even without disease penetrance) have an impact on brain function and organization (Meyer-Lindenberg, 2009), we hypothesized that unaffected relatives with abnormal TDTs would exhibit disordered resting state connectivity in areas involved in the pathophysiology of CD. Abnormal putaminal structure (hypertrophy by voxel based morphometry) and function (reduced activation by fMRI during a temporal discrimination task) have been reported in unaffected relatives with abnormal TDTs (Bradley et al., 2009; Termsarasab et al., 2016). Thus, the assessment of neural connectivity abnormalities associated with abnormal temporal discrimination using rs-fMRI in unaffected first-degree relatives of CD patients may help understand its pathophysiology.

While task-specific neuroimaging studies have great potential to explore the neural basis underlying temporal discrimination and CD, differences found with task-fMRI could reflect compensatory effects of a specific task, thus confounding the primary pathophysiological mechanisms involved. Rs-fMRI data offers a means of observing how functional connectivity relates to human behavior, and how this organization may be altered in neurological diseases (Lee et al., 2013).

We implemented Independent Component Analysis (ICA) in order to explore within network functional connectivity (i.e., functional integration) in the standard resting networks across the two cohorts. In addition to this analysis, we also implemented regional homogeneity (ReHo), and amplitude of low frequency fluctuations (ALFF) analyses in order to study whole-brain local neural activity (i.e., functional segregation) differences between the two cohorts. ALFF measures rs-fMRI signal variability of each voxel in the frequency domain and is considered one of the most reliable and reproducible rsfMRI parameters reflecting the level of regional neural activity (Zang et al., 2007). The ReHo measures the similarity of a given voxel to its neighborhood voxels in the time domain (Zang et al., 2004). ALFF and ReHo have been applied together to reveal different aspects of brain regional function and abnormalities arising in clinical populations (Lei et al., 2012; An et al., 2013; Cui et al., 2014; Premi et al., 2014). Therefore, multiple measures were used in the present study to examine different aspects of the resting state brain in unaffected relatives with normal vs. abnormal TDT, in order to yield a more complete data-driven characterization of resting state whole-brain connectivity (Lv et al., 2018). Based on prior rs-fMRI studies in CD and our hypothesis that unaffected relatives are non-manifesting gene carriers, we postulated that there would be: (1) Significant differences in functional connectivity in the resting state networks previously reported in CD, i.e., the sensory-motor network, the executive control network and the primary visual network; (2) Significant differences in connectivity in other networks relevant to dystonia pathophysiology, i.e., the basal ganglia, midbrain and cerebellar networks; (3) ReHo and power differences across the normal and abnormal TDT cohorts in these regions.

## Materials and Methods

### Temporal Discrimination Threshold Testing

Temporal discrimination testing was carried out and TDT scores were acquired from first degree unaffected relatives of CD patients according to a previously described method (Bradley et al., 2012; Kimmich et al., 2014; Mc Govern et al., 2017). This consisted of (a) Sensory Testing (b) TDT analysis as follows:

#### Sensory Testing

Visual and tactile TDT testing were carried out in a single session, in a sound proof dark room. Asynchronous stimuli were progressively presented to an individual. Participants were tested for two modalities: (i) a visual task (two flashing light-emitting diodes, LEDs); and (ii) a tactile task (non-painful electrical stimulation of the index and middle finger). Stimuli were presented at 5-s intervals and became increasingly asynchronous in steps of 5 ms. The trial ended when the participant reported on three successive instances that the pairs of white LEDs flashed asynchronously. The first of three asynchronous responses was earmarked as the TDT for that trial. This procedure was repeated four times on the left and right side of the body for both modalities (visual and tactile) resulting in a total of 16 runs per participant.

#### Temporal Discrimination Threshold Analysis

All TDT results (in milliseconds) were converted to standardized *Z*-scores using the formula:

$$\mathrm{Z-score}=\frac{\left(\mathrm{actual TDT}-\mathrm{age and gender related control mean TDT}\right)}{\mathrm{Age and gender related control standard deviation}}$$

For each participant, the *Z*-score was calculated using the relevant age-related and gender-related control data set. A TDT value resulting in a *Z*-score ≥ 2.5 was considered an abnormal TDT.

### Participants

**FIGURE 1:**
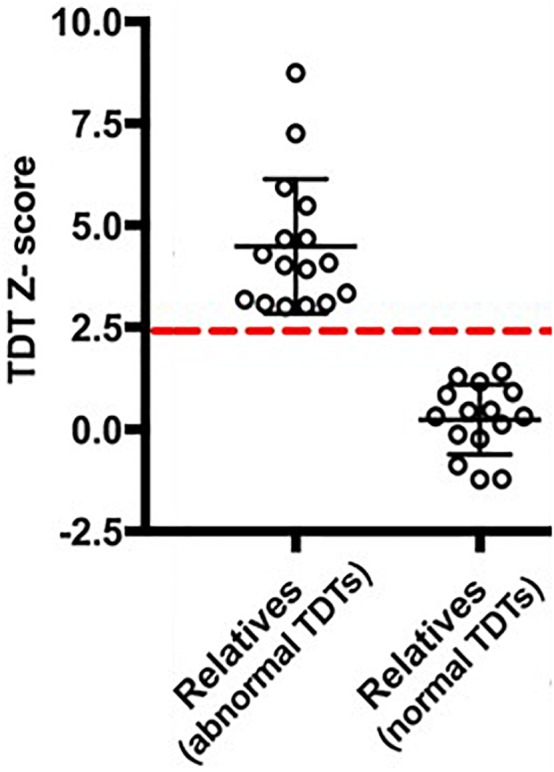
Temporal Discrimination Threshold (TDT) *Z*-scores of the participants. The dashed-red line denotes a TDT *Z*-score of 2.5.

In order to examine any resting state connectivity differences between unaffected relatives with normal TDT versus those with an abnormal TDT, the study was planned to have an equal representation from the two groups; a stratified sample of participants was used where they differed in their TDT values as shown in Figure 1. A total of 32 unaffected relatives were recruited for the rs-fMRI acquisition, according to their TDT scores (Figure 1). Of these, 16 (11 females) had normal TDTs (Relatives_normal TDT_) with a group mean TDT *Z*-score of 0. 22(±0.91) (ranging from −1.23 to 1.4) and 16(11 females) had abnormal TDTs (Relatives_abnormal TDT_) with a group mean TDT *Z*-score of 4.62(±1.69) (ranging from 2.97 to 8.4). The mean age of all 32 relatives was 51.75(±8.11); there was no significant difference in age and gender between the two groups (Table 1). The participants had no history of any neurological or medical condition and all provided a written informed consent. The study was approved by the Medical Research Ethics Committee at St. Vincent’s University Hospital, Dublin.

**Table 1:** Participant demographics.

Subjects	Unaffected relatives with normal TDT	Unaffected relatives with abnormal TDT
Number of participants	16	16
Age (years; mean ± standard deviation)	50.93 ± 7.68	52.75 ± 8.85
TDT (mean ± standard deviation)	0.22 ± 0.91	4.62 ± 1.69
Gender (Female/Male)	11:5	11:5

### MRI Acquisition Protocol

All participants were scanned on a Philips 3T Achieva scanner equipped with a 32 channel head array coil for image acquisition. High-resolution 3D structural scans were acquired using a T1-weighted Magnetization Prepared Rapid Acquisition Gradient Echo protocol. For structural imaging, a TR of 8.4 ms was used along with a TE of 3.9 ms, a flip angle of 8°, transverse orientation, a 256 × 256 matrix size and 0.9 mm isotropic voxels. Resting functional images were collected using 40 slices covering the whole brain (slice thickness 3 mm, inter-slice distance 0 mm, in-plane resolution 3 × 3 mm) with an echo planar imaging (EPI) sequence (TR = 2 s, TE = 25 ms, flip angle = 90°). The resting state scan lasted ∼5.30 min and involved an eyes open fixation on a white cross on a black screen. Restrictive padding was placed around the participant’s head to minimize major head movements.

### Data Pre-processing

Occurrence of motion artifacts was assessed across all acquired images. Mean ± standard deviation of root mean square motion values were as follows: Relatives_normal TDT_ (0.48 ± 0.23) and Relatives_abnormal TDT_ (0.39 ± 0.55). Since no significant differences were found in a 2-sample *t*-test with each subject’s motion values, all scans were included in subsequent analysis. This was followed by the removal of the first four volumes of the rs- fMRI scans in each subject to avoid T1 stabilization effects, high pass filtering (cutoff frequency 0.01 Hz), motion correction, and extraction of non-brain tissue from the whole head images. The functional images were then co-registered to their corresponding anatomical scan using a 6-parameter rigid transformation, normalized to the standard Montreal Neurological Institute T1 2 mm template and further optimized using a non-linear normalization algorithm with a warp resolution of 10 mm. Resampling (4 mm) and variance normalization of the time courses was then carried out. All pre-processing was performed in SPM12^1^ using MATLAB^®^ 2015b (Mathworks Inc.) and FSL v.5.0.4 packages (Jenkinson et al., 2012).

#### Analysis 1: Independent Component Analysis and Dual Regression

To examine large-scale functional connectivity patterns within the standard resting state networks across the two cohorts, we employed ICA and dual regression on the processed data. The pre-processed time series were decomposed into independent components using a multi-session temporal concatenation approach with the MELODIC (Multivariate Exploratory Linear Optimized Decomposition into Independent Components) tool within FSL (Jenkinson et al., 2012). This group-ICA resulted in 41 independent components that were visually examined along with their frequency and time courses. They were also spatially correlated with standard resting state networks (Smith et al., 2009) selected from Smith et al. (2009), using the *Fslcc* utility in FSL to extract the networks relevant to dystonia pathophysiology for further analysis. The components corresponding to the networks relevant to dystonia pathophysiology, i.e., the sensory-motor, executive control and primary visual networks were extracted into one group for a *hypothesis based analysis*, and those corresponding to the midbrain, basal ganglia and cerebellar networks were extracted into another group for an *exploratory analysis*. To assess between-group differences, dual regression was implemented on the set of spatial maps generated from the group-ICA. Dual regression is a reliable and robust mathematical back reconstruction method that utilizes the independent component maps as network templates to identify the corresponding functional connectivity maps in each subject (Beckmann et al., 2009). It was implemented using FSLv.5.0.4. A standard general linear model was designed with additional covariates for the six motion regressors, age and gender. This general linear model was input to a non-parametric permutation method for inference thresholding using the *randomize* function in FSL. The different component maps of each subject were assembled into a 4D file/subject and tested voxel-wise for significant differences (Relatives_abnormal TDT_ > Relatives_normal TDT_ and Relatives_abnormal TDT_ < Relatives_normal TDT_) by performing 5000 random permutations using a threshold free cluster enhanced (TFCE) method to control for multiple comparisons (Smith and Nichols, 2009). This was carried out separately for the hypothesis based analysis and for the exploratory analyses. The statistical significance was set at *p* ≤ 0.05 after family wise error (FWE) correction for multiple comparisons over the component of interest. In addition, Bonferroni correction for the number of resting state networks tested (three networks each in the hypothesis based analyses and exploratory analysis), resulted in a statistical threshold of *p* < 0.017.

#### Analysis 2: Regional Homogeneity (ReHo)

The pre-processed (normalized but unsmoothed) images were subjected to a detrending (linear trend subtraction) and temporal filtering (0.01–0.08 Hz) procedure on the time series of each voxel to reduce the effect of low frequency drifts and high-frequency noise. This was carried out with the REST suite (Song et al., 2011). Following this, ReHo analysis was executed for each participant by computing the Kendall Coefficient of Concordance of the time series of a given voxel with those of its nearest neighbors (18 voxels) at a voxel-wise level. For normalization, each ReHo map was divided by its Kendall global mean value. The data was smoothed with a Gaussian filter of 6 mm full width at half-maximum, to reduce noise and residual differences in gyral anatomy. Age and gender were added as covariates. In order to observe the spatial patterns of each group’s brain ReHo distribution, a one-sample *t*-test (*p* < 0.001) was performed to identify brain regions where the Kendal coefficient was greater than 1. Voxel-wise two-sample *t*-tests were employed to evaluate between group differences. The between-group difference was reported at a corrected *p* < 0.05 (voxel *p* < 0.005 and cluster size >534 mm^3^), using the AlphaSim method as part of the REST suite.

#### Analysis 3: Amplitude of Low Frequency Fluctuations (ALFF)

The previously obtained detrended and filtered signals (from Analysis 2) were used to calculate the amplitude of the low frequency fluctuations (a measure of each voxel’s signal intensity) with the REST suite (Zang et al., 2007). Briefly, this involved converting the time courses to the frequency domain and computing the square root of the power spectrum, followed by averaging across 0.01–0.08 Hz at each voxel. The averaged square root was taken as the ALFF and a normalized map for each voxel was obtained for each subject. Following smoothing with a 6 mm Gaussian filter, a two-sample *t*-test was implemented to determine the ALFF differences between the normal vs. abnormal TDT cohorts with age and gender as covariates. The between-group difference was reported at a corrected *p* < 0.05 (voxel *p* < 0.005 and cluster size >534 mm^3^), using the AlphaSim method as part of the REST suite.

## Results

### Analysis 1: ICA and Dual Regression

#### Hypothesis-Based Analysis

**FIGURE 2:**
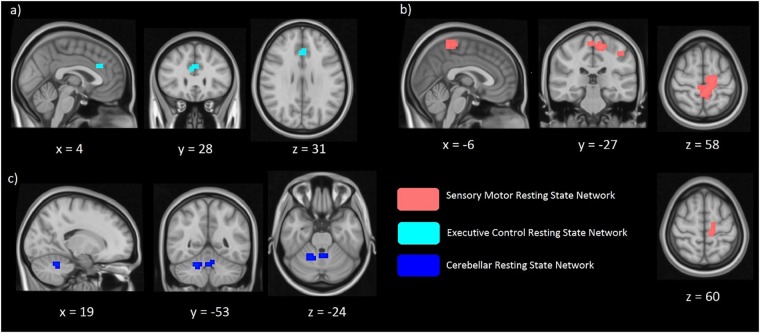
Significant differences of functional connectivity (FC) within the executive control network **(a)** sensorimotor network **(b)**, and cerebellar network **(c)** between relatives with normal vs. abnormal TDT values. Relatives_abnormalTDT_ demonstrated increased FC within the executive control (*p* < 0.017) and cerebellar networks (*p* < 0.05) but decreased FC within the sensorimotor network (*p* < 0.017).

Differences in the spatial distribution of the functional connectivity maps in the sensorimotor, executive controls and visual RSNs were previously found in CD patients when compared to controls (Delnooz et al., 2013; Li et al., 2017). Therefore, these networks formed our hypothesis-based analysis in the two cohorts of unaffected relatives. Significant between-group differences (FWE corrected *p* < 0.017) in the spatial distribution of the functional connectivity maps were found in the sensory-motor and the executive control networks (Figure 2a,b). Relatives with abnormal TDT demonstrated an increase in connectivity in the left and right Brodmann Area (BA) 32 and right BA 8. While BA 32 is the dorsal region of the cingulate gyrus and is associated with rational thought processes and reaction times, BA 8 is involved in planning complex movements. Relatives with abnormal TDT demonstrated a decrease in connectivity in the right and left primary motor areas, left primary sensory area and left sensory associative regions. Contrary to our hypothesis, no significant difference in the spatial distribution of the functional connectivity maps in the visual network was observed.

#### Exploratory-Based Analysis

Due to the cerebellum’s role in temporal perception, balance and it’s implication in the dystonia network, we further explored functional connectivity differences in the cerebellum, derived from Smith et al. (Beckmann et al., 2009), applying a less stringent inference of *p* < 0.05. We also investigated in a similar way the midbrain and basal ganglia networks. The exploratory analysis revealed functional connectivity differences between the abnormal and normal TDT groups for the cerebellar network corresponding to a less stringent threshold of *p* < 0.05 FWE-corrected without Bonferroni correction, as it did not survive the Bonferroni corrected *p* < 0.017 (Figure 2c). However, no significant differences were found between these cohorts for the midbrain and basal ganglia networks.

### Analysis 2: ReHo

**FIGURE 3:**
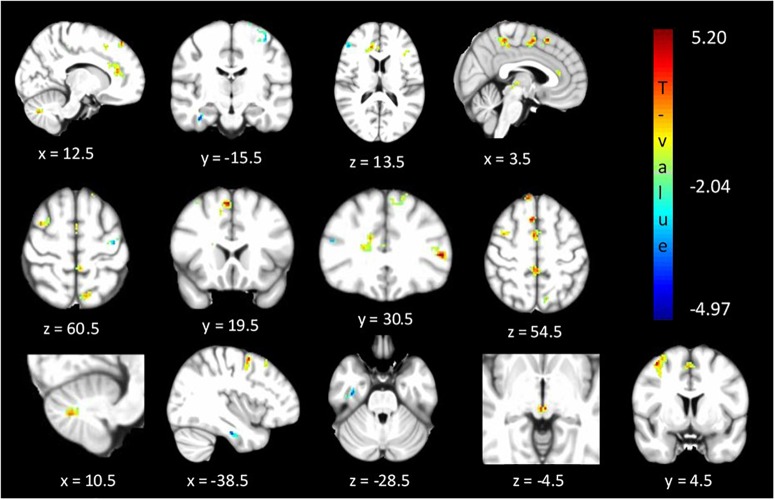
Significant differences (corrected *p* < 0.05) in ReHo values between relatives with normal vs. abnormal TDT values. The blue/green colors represent regions where Relatives_abnormalTDT_ have decreased local coherence than Relatives_normalTDT_. The red/yellow colors represent regions where Relatives_abnormalTDT_ have increased local coherence than Relatives_normalTDT_.

The between cohorts ReHo analysis revealed significant differences (corrected *p* < 0.05) in ReHo values for the cerebellum, thalamus, primary auditory, primary sensory and Brodmann areas as described in Table 2 and shown in Figure 3. Relatives with abnormal TDT demonstrated a decrease in ReHo compared to relatives with normal TDT in the left and right BA 8 (corroborating the findings of the ICA results), in the parahippocampal gyrus and in the left BA 10. Relatives with abnormal TDT demonstrated an increase in ReHo compared to relatives with normal TDT in the thalamus, cerebellum and BA 45 responsible for motor inhibition.

**Table 2:** Significant differences in regional homogeneity (ReHo) (corrected *p* < 0.05) between relatives with normal and abnormal TDT.

*x* (mm)	*y* (mm)	*z* (mm)	Region	Side	Function
**Relatives abnormal TDT < Relatives normal TDT**					
−7	20	55	BA 8	L	FEF-control of eye movements
−30	56	**−**6	BA 10	L	Motor Planning
−39	**−**16	61	BA 6	L	Motor sequencing and planning movements, saccades
36	**−**15	**−**21	ParaHippocampal Gyrus	R	Scene recognition
48	38	14	BA 46	R	Motor planning, organization and regulation, Saccades
12	41	52	BA 8	R	FEF-control of eye movements
**Relatives abnormal TDT > Relatives normal TDT**					
13	**−**70	38	Cerebellum	R	Movement, coordination, precision, timing, balance
12	44	8	BA 10	R	Motor planning
4	**−**36	53	Sensory Associative area	R	Visuospatial, visuo-motor processing, saccades
−7	20	55	BA 8	R	FEF-control of eye movements
−3	**−**23	**−**1	Thalamus	L/R	Sensory perception, motor regulation
5	**−**1	53	BA 6	R	Motor sequencing and Planning movements, saccades
−2	**−**26	**−**5	Brainstem and Midbrain	L/R	Control, conduction, multisensory integration
−49	31	7	BA 45	L	Motor inhibition
−12	**−**63	61	BA 7	L	Visual-motor coordination, motor execution, saccades

*The columns denote the clusters’ x, y, z coordinates in mm, the area represented by them, side of the brain and function of the region. Clusters with voxel p < 0.005 and size >534 mm^3^ have been reported.*

### Analysis 3: ALFF

**FIGURE 4:**
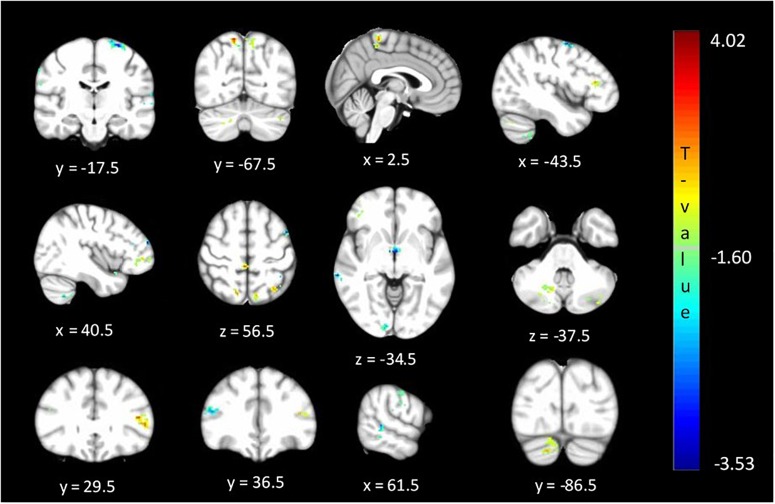
Significant differences (corrected *p* < 0.05) in ALFF values between relatives with normal vs. abnormal TDT values. The blue/green colors represent regions where Relatives_abnormalTDT_ have decreased signal power than Relatives_normalTDT_. The red/yellow colors represent regions where Relatives_abnormalTDT_ have increased signal power than Relatives_normalTDT_.

The between cohort analysis revealed significant differences (corrected *p* < 0.05) in the ALFF between the two cohorts of unaffected relatives in the cerebellum, hypothalamus, primary visual, primary sensory and Brodmann areas as described in Table 3 and shown in Figure 4. Relatives with abnormal TDT demonstrated a decrease in ALFF in the primary visual area, hypothalamus, BA 46 related to saccadic activity and left primary auditory area. However, there was a decrease in BA 7, BA 45 and the sensory associative regions.

**Table 3:** Significant differences in ALFF differences (corrected *p* < 0.05) between relatives with normal and abnormal TDT.

*x* (mm)	*y* (mm)	*z* (mm)	Region	Side	Function
**Relatives abnormal TDT < Relatives normal TDT**					
42	36	16	BA 46	R	Motor planning, organization and regulation, Saccades
42	53	20	BA 10	R	Motor planning
−29	**−**18	67	BA 6	L	Motor sequencing and planning movements, saccades
11	**−**95	**−**6	Primary Visual	R	Visual processing
−3	**−**3	**−**5	Hypothalamus	L/R	Emotions, temperature
62	**−**35	2	BA 21	R	Visual, auditory, deductive reasoning, language
34	**−**71	**−**38	Cerebellum	R	Movement, coordination, precision, timing, balance
−67	**−**22	7	Primary Auditory	L	Auditory information processing
**Relatives abnormal TDT > Relatives normal TDT**					
−33	**−**61	57	BA 7	L/R	Object spatial location
3	**−**35	62	Primary Motor	R	Movement execution
3	**−**34	53	Sensory Associative	R	Integration of sensory information, memory, learning
−51	30	7	BA 45	L	Movement, response inhibition, memory
15	**−**87	**−**34	Cerebellum	R/L	Movement, coordination, precision, timing, balance

*The columns denote the clusters’ x, y, z coordinates in mm, the area represented by them, side of the brain and function of the region. Clusters with voxel p < 0.005 and cluster size >534 mm^3^ have been reported.*

## Discussion

This study explored the neural connectivity basis of abnormal temporal discrimination as an endophenotype for CD. The findings revealed altered intrinsic regional activity in unaffected relatives of CD patients, who have abnormal temporal discrimination. These connectivity differences were measured by studying within network metrics via ICA and Dual Regression analyses, as well as ReHo and signal fluctuation amplitude analyses. As part of the hypothesis based analysis, our results demonstrated altered resting state functional connectivity in the sensory-motor (decreased connectivity) and executive control (increased connectivity). As part of the exploratory-based analysis, or results demonstrated altered resting state connectivity in the cerebellar (increased connectivity) network in relatives with abnormal TDT when compared to relatives with normal TDT values. Areas related to motor planning, motor learning, visual motion processing, saccades, emotion and visual-motor coordination were observed to have local functional incongruities between the two groups. An abnormal TDT in unaffected first-degree relatives was found to be linked with local brain function abnormalities in regions associated with CD pathophysiology. This would be consistent with the hypothesis that these relatives are non-manifesting dystonia gene carriers.

Event related fMRI studies have examined the time course of activation associated with different components of a time perception (Rao et al., 2001; Nenadic et al., 2003; Pastor et al., 2004). The results of these studies demonstrated a dynamic and complex network of cortical and subcortical activation associated with different components of temporal processing, implicating the basal ganglia, cerebellum and sensory-motor regions in the tasks involved. We observed similar patterns of differential brain function in these areas when comparing relatives with abnormal and normal TDTs.

**FIGURE 5:**
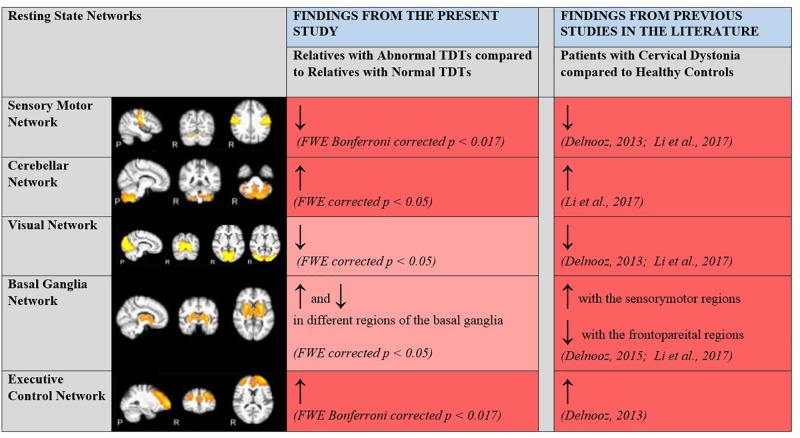
Changes in within network functional connectivity in relatives with abnormal TDT when compared to relatives with normal TDT. Comparative analyses with previous rsfMRI connectivity studies carried out with patients with CD and healthy controls. ↑, Increased connectivity; ↓, Decreased connectivity; Dark Red, Significant changes in functional connectivity, ReHo and ALFF; Light Red, No significant changes in functional connectivity detected via the ICA method but significant changes in regional coherence (ReHo) and power (ALFF) detected. Resting State Networks shown adapted from Castellazzi et al. (2014).

A rs-fMRI study, carried out with CD patients and controls (Delnooz et al., 2013; Li et al., 2017), established within-network functional connectivity differences in the sensory-motor, executive control and primary visual networks. CD patients showed a decrease in connectivity within the sensory-motor network and an increase in connectivity within the executive control network compared to controls. Since these areas could also be expected to be involved in the visual temporal discrimination task, and in keeping with the hypothesis that both CD and abnormal TDT arise from common defective cortical and sub-cortical mechanisms, it was hypothesized that there would also be within-network functional connectivity differences in these networks (sensory-motor, executive control and primary visual) across abnormal vs. normal TDT subjects. Consistent with our hypothesis and previous literature, connectivity differences were found in the sensory-motor and executive control networks. Relatives with abnormal TDTs demonstrated decreased connectivity in the sensory-motor network compared to relatives with normal TDTs. For the executive control network, relatives with abnormal TDTs demonstrated an increase in connectivity compared to relatives with normal TDTs. This was complemented by results from the ReHo and ALFF analyses. Together the results from the present and previously reported studies (Figure 5), suggest that the CD and temporal discrimination circuit share common dysfunctional nodes. This cautiously suggests that TDT may indeed be a true endophenotype of dystonia (given the finding of similar abnormalities), while, on the other hand, the data does not indicate the actual origin of the abnormal TDT itself. Although a causal/consequential role cannot be determined at this stage, the presence of neural connectivity related changes in unaffected relatives with abnormal TDTs supports the concept of the TDT as an endophenotype in this disorder. Network-related changes have been observed in non-manifesting carriers of DYT1 mutations in the posterior putamen/globus pallidus, cerebellum and sensory motor area and thus were unrelated to the presence of symptoms (Tros et al., 2002; Carbon et al., 2004). Our results also suggest that the observed network related changes are not due to dystonia manifestation, but relate instead to the altered neural mechanisms caused by poorly penetrant genetic mutations.

A fundamental element of sensorimotor function is the accurate discernment and organization of the temporal characteristics of sensory stimuli and motor output. Optimal movement execution entails precise processing of sensory information from the environment, as is evidenced from visuo-motor, touch, and proprioception experiments (Tinazzi et al., 2013; Tinazzi et al., 2014). In the present study, the presence of local sensory-motor connectivity discrepancies in unaffected relatives with abnormal TDTs points to the existence of abnormal connectivity patterns in the sensory motor and executive control areas, prior to disease manifestation. Future studies involving varied sensory-motor tasks in unaffected relatives need to be carried out, in order to probe the extent of involvement and manifestation of the sub-clinical sensory-motor and executive control abnormalities.

The cerebellum is also involved in a complex network for sensory-motor integration (Brunamonti et al., 2014). Due to the role of the cerebellum in timing related events (Manganelli et al., 2013; Manto et al., 2013) and the increasing evidence of the cerebellum’s role in dystonia (Prudente et al., 2014), exploratory analysis was carried out to study resting state connectivity in this region. The results revealed significant differences in connectivity within the cerebellum between subjects with abnormal and normal TDTs; those with abnormal TDTs showed an increase in functional connectivity compared to those with normal TDTs. This was corroborated by higher signal power (ALFF) and local coherence (ReHo) in the area. Studies have reported diminished time perception in patients with cerebellar deficits (Di Biasio et al., 2015; Raghavan et al., 2016). The bulk of cortical input to the cerebellum (via the pons) comes from the sensory and parietal areas (Pastor et al., 2004). Time perception deficits have been correlated with abnormalities in the vermis, as well as the lateral cerebellar hemispheres (Rao et al., 2001). The activation of the cerebellum in both spatial and temporal discrimination tasks using tactile stimuli in an fMRI study, suggested its role in optimizing the perception of sensory inputs (Pastor et al., 2004). Our results corroborate past neuroimaging research with these temporal discrimination tasks and cerebellar atrophy studies (Manganelli et al., 2013). Furthermore, our results point to the presence of cerebellar deficits in unaffected first-degree relatives with abnormal TDT, which could be a substrate for eventual CD manifestation (which may depend on environmental insults). The presence of increased as well as decreased ALFF values in different regions of the cerebellum may indicate the development of compensatory mechanisms in unaffected relatives.

In an fMRI study exploring temporal information processing and the basal ganglia (Nenadic et al., 2003), time estimation (vs. rest condition) elicited a distinct pattern of activity in the right medial and both left and right dorsolateral prefrontal cortices, thalamus, basal ganglia (caudate nucleus and putamen), left anterior cingulate cortex, and superior temporal auditory areas. The thalamus and putamen have been considered as important nodes in the temporal discrimination circuit (Lacruz et al., 1991). Some neural models of temporal information processing consider that fronto-subcortical circuits linking the prefrontal cortical areas with the striatum and thalamus have a specific role in temporal processing (Gibbon et al., 1997). Our results likewise demonstrated increased ReHo in the thalamus in relatives with abnormal TDTs. The absence of connectivity related differences in the visual, basal ganglia and midbrain networks, as measured by the independent component analyses, indicates the non-existence of the manifestation of abnormalities in these regions in relatives. Further, it could also be due to visual related connectivity differences manifesting only during task activation or the need for specific region of interest masks for more precise identification (Delnooz et al., 2015). Regional connectivity changes in these areas were however detected via ReHo and ALFF metric abnormalities between the two groups. The involvement of areas related to motor planning, learning, movement coordination, saccades, executive function, cross-modal association and emotion, suggests a multi-network dysfunction manifestation due to (as yet unidentified) gene carriage in unaffected relatives of CD patients. Dysfunction in these regions is consistent with CD pathophysiology (Muller et al., 2005; Chillemi et al., 2017) and our results thus support the plausible concept of susceptibility gene carriage in these unaffected relatives.

The primary limitation of the current study is that it cannot be ascertained whether the abnormalities detected represent the effects of a pre-symptomatic phase due to expression of an abnormal gene or a protective neuroplastic response to that gene. The number of subjects in each group is quite low. Future studies looking to replicate these findings could aim for a larger cohort of relatives and extend the findings to comparisons between patients and controls.

## Conclusion

In the present study, the neural circuitry of disordered temporal discrimination was studied in the context of its role as an endophenotype in CD. Our results demonstrated significant differences in resting functional connectivity in the sensory-motor, executive control and cerebellar networks, in relatives with abnormal TDTs when compared to relatives with normal TDTs. The aberrations detected in the present study occurred in regions previously associated with the pathophysiology of CD. They suggest possible resting brain function alterations that may be an indicator of neural plasticity or impending disorder manifestation, caused by the carriage of, as yet unidentified, genes in unaffected first degree relatives with abnormal TDT. The results collectively: (1) Indicate a multi-network system level dysfunction in relatives with abnormal TDTs involving hubs associated with CD. (2) Demonstrate that this occurs by virtue of regional changes (markers of dysfunction – connectivity, homogeneity and frequency power) in these networks. (3) Support the hypothesis that the manifestation of this network dysfunction in unaffected relatives is due to the carriage of as yet unidentified and poorly penetrant genes. (4) Provide evidence that unaffected relatives with abnormal TDTs have certain network aberrations, which, following appropriate environmental exposures may result in disease penetrance. A longitudinal analysis carried out by studying relatives with abnormal TDTs who later go on to manifest dystonia, would strengthen the clinical value of abnormal TDT as an indicator of as yet unidentified genes, as well as its utility as a predictive marker of the eventual disease manifestation.

## Data Availability

Data and material used in the present study will be made available upon request to interested researchers.

## Author Contributions

SN, MH, and RR conceived the research. SN, EMcG, MH, and RR organized the research project. SN executed the research and statistical analysis, and wrote the first draft of the manuscript. SN and RR designed the statistical analysis. SO’R, MH, and RR reviewed and critically assessed the statistical analysis. EMcG, RB, SO’R, MH, and RR reviewed and critically revised the manuscript. All authors reviewed the manuscript.

## Conflict of Interest Statement

The authors declare that the research was conducted in the absence of any commercial or financial relationships that could be construed as a potential conflict of interest.

## Footnotes

1 http://www.fil.ion.ucl.ac.uk/spm/software/spm12/
